# Single-Cell RNA Sequencing Reveals B Cells Are Important Regulators in Fracture Healing

**DOI:** 10.3389/fendo.2021.666140

**Published:** 2021-11-08

**Authors:** Hao Zhang, Renkai Wang, Guangchao Wang, Bo Zhang, Chao Wang, Di Li, Chen Ding, Qiang Wei, Zhenyu Fan, Hao Tang, Fang Ji

**Affiliations:** ^1^ Department of Orthopedics, Changhai Hospital, Secondary Military Medical University, Shanghai, China; ^2^ Guangdong Key Lab of Orthopedic Technology and Implant Materials, Key Laboratory of Trauma and Tissue Repair of Tropical Area of People’s Liberation Army (PLA), Hospital of Orthopedics, General Hospital of Southern Theater Command of People’s Liberation Army, Guangzhou, China; ^3^ Department of Bioinformatics, Novel Bioinformatics Ltd., Co., Shanghai, China; ^4^ Department of Orthopedics, The Ninth People’s Hospital, Shanghai Jiaotong University, Shanghai, China

**Keywords:** scRNA-seq, fracture healing, B cells, exosome, bone marrow

## Abstract

The bone marrow microenvironment is composed primarily of immune and stromal cells that play important roles in fracture healing. Although immune cells have been identified in mouse bone marrow, variations in their numbers and type during the fracture healing process remain poorly defined. In this study, single-cell RNA sequencing was used to identify immune cells in fracture tissues, including neutrophils, monocytes, T cells, B cells, and plasma cells. The number of B cells decreased significantly in the early stage of fracture healing. Furthermore, B cells in mice fracture models decreased significantly during the epiphyseal phase and then gradually returned to normal during the epiphyseal transformation phase of fracture healing. The B-cell pattern was opposite to that of bone formation and resorption activities. Notably, B-cell–derived exosomes inhibited bone homeostasis in fracture healing. In humans, a decrease in the number of B cells during the epiphyseal phase stimulated fracture healing. Then, as the numbers of osteoblasts increased during the callus reconstruction stage, the number of B cells gradually recovered, which reduced additional bone regeneration. Thus, B cells are key regulators of fracture healing and inhibit excessive bone regeneration by producing multiple osteoblast inhibitors.

## Introduction

Bone fractures are the most common bone traumatic diseases worldwide (1). Severe multiple fractures caused by car accident or fall injury are usually accompanied by delayed healing and even nonunion (2). Fracture healing is directly regulated by the function and number of osteoblasts and osteoclasts (3, 4). However, numerous risk factors affect bone repair, including age, infection, malnutrition, distribution of blood vessels, activation of immune response, and inappropriate fracture fixation (5, 6). In the initial inflammatory stage, necrotic tissues are removed, and specific immune cells create a suitable bone marrow microenvironment for fracture healing. Notably, the immune system is suppressed by fracture, and an increase in T regulatory cells inhibits active adaptive immune responses (7). Furthermore, mesenchymal stem cells maintain a hypoimmunogenic state, revealing that immune cells can inhibit fracture healing (8). However, the effects of immune cells on fracture healing have yet to be fully determined.

Several cytokines or other factors are essential for B cell development, including the receptor activators of NF-κB ligand (RANKL), OPG, IL-7, and CXCL-12, which are secreted by bone marrow stromal cells or osteoblasts (9, 10). Furthermore, B cells themselves can secret RANKL, suggesting that B cells can affect the differentiation of osteoclasts (11). In fact, mice lacking RANKL in B lymphocytes are partially protected from bone loss associated with ovariectomy. However, in mice with conditional knockout of RANKL in T lymphocytes, there is no effect on such bone loss (11). Moreover, B cells can inhibit bone formation in rheumatoid arthritis by suppressing osteoblast differentiation (12), which reveals that B cells have an important role in osteoimmunology. However, owing to the complexity of the bone marrow microenvironment in fracture healing, the effect of B cells on fracture healing remains unclear.

Single-cell RNA sequencing (scRNA-seq) has recently revolutionized study of the bone marrow microenvironment (13). With this approach, individual single cells can be clustered by transcriptome analysis rather than by surface markers. Furthermore, numerous cell types can be characterized between control and case samples, which helps to comprehensively understand the heterogeneity of diseases, including cancers, inflammation, and infection, among others (14, 15). During fracture healing, multiple cell types are involved in the bone marrow microenvironment, including immune cells, endothelial cells, hematopoietic stem cells, bone marrow stromal cells, osteoblasts, and osteoclasts (16). Therefore, to understand the mechanism for delayed fracture healing and bone nonunion, the developmental pseudotime trajectory needs to be constructed and cell–cell interactions determined.

In this study, the focus was on the roles of B cells in fracture healing. The scRNA-seq revealed fewer B cells in a patient with old fracture tissues than in one with fresh fracture tissues. Therefore, a mice fracture model was generated, and scRNA-seq showed that B cells decreased during the epiphyseal phase and increased during the callus reconstruction stage. Exosomes derived from B cells (BC-Exos) affected bone homeostasis *in vitro* by promoting osteoclastic bone resorption and inhibiting osteoblastic bone formation. Furthermore, osteogenic activity was inhibited *in vivo* in mice injected with BC-Exos. Thus, these results reveal that B cells and BC-Exos have important regulatory roles in fracture healing.

## Materials and Methods

### Preparation of Human Fracture Tissues

Following surgical resection, samples from human fracture tissues were collected from two patients with femur fractures in Shanghai Changhai Hospital, Second Military Medical University. The approval for this clinical study was provided by the Committees of Clinical Ethics of Shanghai Changhai Hospital, Second Military Medical University. Informed consent was obtained from the patients.

### Mice

All animal experiments were undertaken in accordance with the National Institute of Health Guide for the Care and Use of Laboratory Animals and with the approval of the Scientific Investigation Board of Second Military Medical University (Shanghai, China). C57BL/6 mice (6 to 8 weeks old) were obtained from Shanghai SLAC Laboratory Animal Co., Ltd. All mice were housed at the animal center of the Second Military Medical University.

For construction of mice fracture models, briefly, after anesthesia and surgical site sterilization, we first cut the skin, fascia, and muscle tissues to expose the femur and patella. Then, we construct transverse femoral fractures by pendulum saw. A Kirschner wire with a diameter of 0.5 mm was then inserted into the bone marrow space to stabilize the fracture. Then, we placed muscle over the osteotomy site and stitched with absorbable sutures prior to closing the skin with wound clips.

For delivery of B Cell Exosomes *in vivo*, 100μl exosomes in 0.1 ml saline buffer was locally injected into tail vein once every 3 days for 2 weeks.

### Isolation of Bone Tissues

To obtain bone and bone marrow cells for scRNA-seq, bone fracture tissues (including bone and bone marrow) were dissected from patients, transported to the research facility, and then placed in DMEM (4.5 g/L, Gibco, USA). The bone fracture tissues were cut into small fragments and digested with 0.2 mg/ml collagenase II (Invitrogen) for 2 h at 37°C. After digestion, the cells were filtered through a 70-μm strainer (Corning) into a collection tube. Erythrocytes were lysed in ACK-lysis buffer (Sigma) for 5 min. Following centrifugation at 1,000 rpm at 4°C for 8 min, the supernatant was decanted and discarded. For further experiments, cells were resuspended in PBS (Gibco) and filtered through a 70-μm strainer on ice.

### Cell Culture

BaF cells were cultured in RPMI-1640 (Hyclone, USA), containing 10% FBS (Thermo, USA), 10 ng/ml IL-3 (PeproTech, USA), and 1% penicillin–streptomycin (Thermo) at 37°C in a 5% CO_2_ humidified incubator.

To induce osteoblast differentiation, BMSC cells were seeded in a 12-well plate with osteogenic differentiation complete medium (including osteogenic differentiation basal medium, 175 ml; osteogenic differentiation fetal bovine serum, 20 ml; penicillin-streptomycin, 2 ml; glutamine, 2 ml; ascorbate, 400 μl; β-glycerophosphate, 2 ml; and dexamethasone, 20 μl) (Cyagen), which was changed every 3 days. At day 21, the cells were stained with Alizarin red (pH 5.5) to analyze osteogenic differentiation.

For osteoclast differentiation, mouse bone marrow monocytes isolated from femurs and tibias were seeded at 1 × 10^6^ cells per well in a 24-well plate. The cells were cultured in DMEM (4.5 g/L, Gibco) supplemented with 10% FBS (Thermo), 50 ng/ml M-CSF (PeproTech, USA), and 1% penicillin–streptomycin (Thermo) for 3 days at 37°C in a 5% CO_2_ humidified incubator. Nonadherent cells were discarded, and the adherent cells were cultured in DMEM (4.5 g/L, Gibco) supplemented with 10% FBS (Thermo), 50 ng/ml M-CSF (PeproTech), 100 ng/ml soluble RANKL (PeproTech), and 1% penicillin–streptomycin (Thermo) for 5 days. The medium was changed every 3 days. At day 8, the cells were stained with TRAP using a commercial kit according to the manufacturer’s instructions (Sigma) (17).

### Isolation of B Cell Exosomes

The exosomes of B cells were extracted as previously described (18). Briefly, BaF cells were cultured in complete medium containing EV-free FBS (BI) for 24 h. Then, the supernatant containing BC-Exos was centrifuged at 2,000 rpm for 30 min to eliminate dead cells and cellular debris. The supernatant containing the cell-free culture media was transferred to a new tube, and 0.5 volumes of the Total Exosome were added.

The culture media and an isolation reagent (Thermo) were mixed by vortexing or by pipetting up and down until a homogenous solution was obtained. The solution was incubated at 2°C to 8°C overnight. After incubation, the samples were centrifuged at 10,000×*g* for 1 h at 2°C to 8°C. The supernatant was aspirated and discarded, and exosomes were contained in the pellet at the bottom of the tube. The pellet was resuspended in 1× PBS. Protein content of BC-Exos was determined by using a BCA Protein Assay Kit (Thermo Fisher Scientific, USA).

### Characterization of B Cell Exosomes

The morphology of BC-Exos was observed with a transmission electron microscope (Hitachi, Japan). The size distribution of BC-Exos was determined using a Nanosizer™ (Malvern Instruments) (18). The expression of surface markers (CD9, CD63) on BC-Exos was identified with Western blot (WB).

### Immunofluorescence Staining

Fresh bone tissues dissected from mice fracture models were fixed in 4% paraformaldehyde overnight. Tissues were decalcified with 0.5 M EDTA with constant shaking, and then, bone tissues were embedded in OCT (Sakura). Bone sections, 6 μm, were stained with antibodies B220 (1:100, Abcam) and osteocalcin (1:100, Abcam). Then, secondary antibodies conjugated with fluorescence (1:100, Jackson) were used, and nuclei were counterstained with DAPI. Bone tissues were observed under a confocal microscope.

### Reverse-Transcription Quantitative PCR

Total RNA from cultured cells was prepared using TRIzol reagent (Thermo) and then reverse-transcribed into cDNA with a cDNA reverse-transcription kit (Applied Biosystems, USA). Reverse-transcription quantitative PCR (RT-qPCR) was performed with an ABI Prism 7900 HT Sequence detection system (Applied Biosystems). The primer sequences used in the RT-qPCR were as follow: Alp: forward, 5′-CCA ACT CTT TTG TGC CAG AGA-3′; reverse, 5′-GGC TAC ATT GGT GTT GAG CTT TT-3′; BGLAP: forward, 5′-CTG ACC TCA CAG ATG CCA AGC-3′; reverse, 5′-TGG TCT GAT AGC TCG TCA CAA G-3′; Ctsk: forward, 5′-CTC GGC GTT TAA TTT GGG AGA-3′; reverse, 5′-TCG AGA GGG AGG TAT TCT GAG T-3′; and TRAF-6: forward, 5′-AAG GTG GTG GCG TTA TAC TGC-3′; reverse, 5′-CTG GCA CAG CGG ATG TGA G-3′ (4).

### μCT Analysis

Femur samples were dissected from mice and analyzed through micro-CT machine (Quantum GX, PE). We performed micro-CT scans under the same conditions: voltage 120 kV, current 60 mA, spatial resolution 10 mm, scanning 1000 continuous sections. Then the data was collected and analyzed automatically to analyze the number of trabecular bones (Tb.N), trabecular bone thickness (Tb.Th), trabecular bone space (Tb.Sp), bone volume fraction (BV/TV), and other indicators.

### Single-Cell RNA Sequencing Experiment

The transcriptomic information of single cells was captured by using the BD Rhapsody system. Single-cell capture was achieved by random distribution of a single-cell suspension across >200,000 microwells through a limited dilution approach (13), Which fracture tissues were dissected and cut into small pieces. Then, they were digested by 3 mg/ml protease (thermo, usa) for 60 minutes and 2 mg/ml collagenase P (thermo, USA) for 2 to 3 h. Beads with oligonucleotide bar codes were added to saturation so that a bead was paired with a cell in a microwell. Cell-lysis buffer was added to hybridize poly-adenylated RNA molecules to the beads. Beads were collected into a single tube for reverse transcription. After cDNA synthesis, each cDNA molecule was tagged on the 5′ end (that is, the 3′ end of a mRNA transcript) with a unique molecular identifier (UMI) and cell label indicating its cell of origin. Whole transcriptome libraries were prepared using the BD Rhapsody single-cell whole-transcriptome amplification workflow. In brief, second-strand cDNA was synthesized, followed by ligation of the WTA adaptor for universal amplification. Eighteen cycles of PCR amplified the adaptor-ligated cDNA products. Sequencing libraries were prepared using random priming PCR of the whole-transcriptome amplification products to enrich the 3′ end of the transcripts linked with the cell label and UMI. Sequencing libraries were quantified using a High Sensitivity DNA chip (Agilent) on a Bioanalyzer 2200 in a Qubit High Sensitivity DNA assay (Thermo Fisher Scientific). The library for each sample was sequenced by HiSeq Xten (Illumina) in a 150-bp paired-end run.

### Cell Normalization

The Seurat (v 3.0) [https://satijalab.org/seurat/] package was applied according to the cell raw counts calculated by UMI-Tools for cell normalization and cell filtering considering the MT percentage (20% MT expression) and minimum (200) and maximum (5,000) gene numbers. Seurat regression was based on the MT and UMI counts for batch effector removal to achieve scaled data. PCA and t-SNE analysis were used to reduce the dimensions of the highly variable genes (13).

### Cell Clusters and Subclusters

Clusters were identified by a graph-based and k-mean-based clustering approach implemented by the FindCluster function in Seurat [https://satijalab.org/seurat/]. The Wilcox *t*-test was used in the FindAllMarkers function in Seurat to discover the marker genes of each cluster.

### Cell Communication

To enable a systematic analysis of cell–cell communication molecules, cell communication analysis was based on the CellPhoneDB (19), a public repository of ligands, receptors, and their interactions. The membrane, secreted, and peripheral proteins of a cluster at different time points were annotated. Significant mean and cell communication significance (*p* < 0.05) were calculated based on the interaction and the normalized cell matrix achieved by Seurat normalization.

### Pseudotime Analysis

In the Seurat package including clustering and cell marker identification, Monocle2 (20) was used for pseudotime analysis. The state of cell processing was analyzed, and single cells were placed in a cluster along a trajectory according to a biological process, such as cell differentiation, by taking advantage of an individual cell’s asynchronous progression in those processes.

### Single-Cell RNA Statistical Analysis

To obtain clean data, fastp with default parameters was used to filter the adaptor sequences and remove the low-quality reads. UMI-Tools was used in the Single Cell Transcriptome Analysis to identify the cell bar code white list. To obtain the UMI counts of each sample, UMI-based clean data were mapped to the mouse genome (Ensemble v 92) using STAR (21) mapping with customized parameters from the UMI-Tools standard pipeline (22). To minimize the sample batch, down-sample analysis was applied among samples sequenced according to the mean reads per cell of each sample to finally achieve a cell expression table with sample bar codes. Cells that contained over 200 expressed genes and mitochondria UMI rate below 20% passed the cell quality filtering. Mitochondria genes were removed from the expression table but were used for cell expression regression in order to avoid the effects of the cell status on the clustering and marker analyses of each cluster.

The Seurat package (v 2.3.4, https://satijalab.org/seurat/) was used for cell normalization and regression based on the expression table according to the UMI counts of each sample and percent of mitochondria rate to obtain the scaled data. PCA was conducted based on the scaled data with all highly variable genes, and the top eight principals were used for t-SNE construction. A graph-based cluster method was used to acquire the unsupervised cell cluster results on the basis of the top eight principals. The marker genes were calculated by the FindAllMarkers function with the Wilcox rank-sum test algorithm under the following criteria: logFC > 0.25; *p* < 0.05; min. pct. > 0.1. To identify the cell type detailed, osteoblast and osteoclast cells were selected for re-tSNE analysis, graph-based clustering, and marker analysis.

### Statistical Analyses

Data are presented as the mean ± standard deviation (SD). Two-tailed Student’s *t*-test was used to compare means between two groups, and one-way ANOVA was used to compare means between multiple groups. GraphPad Prism Software was used for statistical analyses, with significance at *p < 0.05 and **p < 0.01.

### Data Availability

Single-cell RNA-seq data are available at GEO (Gene Expression Omnibus) under accession numbers GSE142786 and GSE132884.

## Results

### Single-Cell Profiling of Human Fracture Tissue Cells

**Figure 1:**
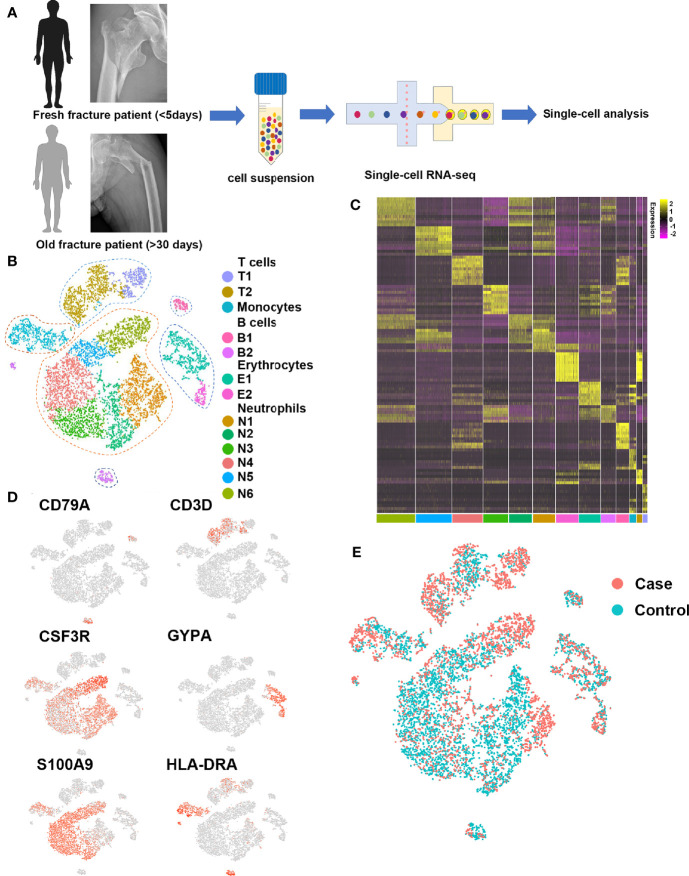
Single-cell profiling of human fracture tissue cells. **(A)** Schematic of the experimental strategy. **(B)** t-SNE visualization of human fracture tissue cells. **(C)** Heat map of the scaled expression of differentially expressed genes for each cluster in human fracture tissue cells. **(D)** Dot plots showing the expression of markers for each cell cluster in human fracture tissue cells. **(E)** t-SNE visualization for old fracture patient (case, red) and fresh fracture patient (control, green).

To isolate human fracture tissue cells at different stages following fracture, fresh fracture tissues (control, less than 5 days) and old fracture tissues (case, more than 30 days) were obtained from two patients that had hip replacement surgery (**Figure 1A**). In total, 9,976 individual cells associated with fracture were sequenced, of which 9,339 were retained after rigorous filtration for subsequent analysis.

To investigate the cell populations that played key roles in fracture healing, 13 cell clusters were identified with *t*-distributed stochastic neighbor embedding (t-SNE) (**Figures 1B, C**), including six neutrophil clusters (expressing CSF3R, ELANE, FCGR3B, MKI67, MPO, MS4A3, and OLR1), two early erythrocyte clusters (expressing GYPA, HBA1, and HBB), two T cell clusters (expressing CD3D, CD3E, CD3G, CD4, CD8, and NKG7), one B cell cluster (expressing CD22, CD79A, and MS4A1), one plasma cell cluster (expressing CD79A, IGHG1, and IGKC), and one monocyte cluster (expressing CD1C, HLA-DRA, ITGAM, and S100A9). Representative markers for neutrophils, early erythrocytes, T cells, B cells, plasma cells, and monocytes were identified (**Figure 1D**).

To investigate differences in the cell populations between fresh and old fracture patients, the cell clusters of the two groups were compared in t-SNE (**Figure 1E**). There were many differences in the amount of B cells. The B cells in the bone marrow microenvironment can affect the differentiation of osteoblasts and osteoclasts (23, 24). Moreover, scRNA-seq showed little or no difference in the numbers of the other immune cells (**Figures S1, S2**), including monocytes, neutrophils, and T cells. Therefore, the focus was on the roles of B cells in fracture healing.

### B Cells in Human Fracture Tissues

**Figure 2:**
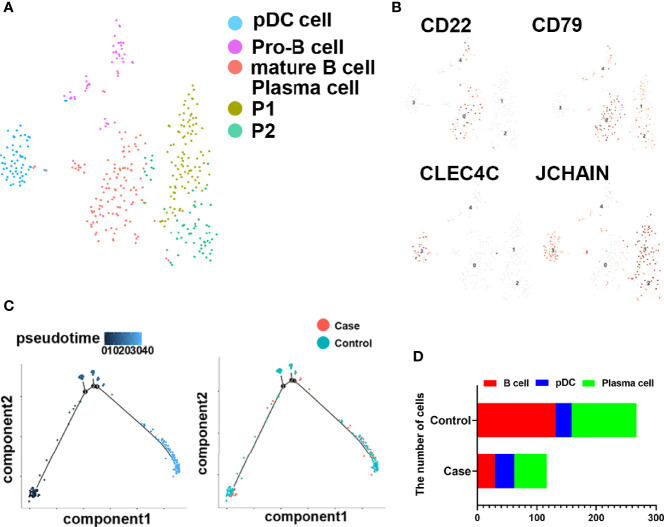
B cells in human fracture tissue. **(A)** t-SNE visualization of B cell subpopulations in human fracture tissues. **(B)** Dot plots showing the expression of markers for each cell cluster in the B cell cluster. **(C)** Monocle2 pseudotime trajectory of the differentiation of B cells in human fracture tissues. **(D)** Composition and proportion of cells in case and control groups.

To investigate the roles of B cells in fracture healing, five populations of B cells in human fracture tissues were defined: pDC cells (expressing CLEC4C), pro-B cells (expressing HLA-DRA and CD74), mature B cells (expressing CD23 and CD24), and plasma cells (including P1 and P2, expressing J-chain). Then, the relationships between B cells and fracture healing were analyzed to identify distinctive cluster markers (**Figures 2A, B**). Furthermore, the ratios among different cells were calculated, and the numbers and proportions of B cells in the case group were less than those in the control group (**Figure 2C**, **Table 1**). However, the pseudotime trajectory axis indicated that the B cell constitution in each period in the case group was comparable with that in the control group (**Figure 2D**), suggesting that all B cell subsets decreased in the stages of hematoma and osteophyte formation. Collectively, the results indicated that B cells decreased significantly in the old fracture, which might affect fracture healing.

**Table 1:** The number of immune cells in the case group and the control group.

Sample	CellType	Percentage	Number
Case	B_Cell	1.93%	74
Case	Early_Erythroblast	11.99%	460
Case	Granulocyte_Progenitor	12.43%	477
Case	Granulocytes	19.36%	743
Case	MDSC	13.34%	512
Case	Monocyte	9.17%	352
Case	NK_Cell	8.76%	336
Case	Plasma_Cell	1.33%	51
Case	Proliferating_Granulocyte_Progenitor	5.71%	219
Case	T_Cell	15.98%	613
Control	B_Cell	2.85%	157
Control	Early_Erythroblast	9.94%	547
Control	Granulocyte_Progenitor	14.36%	790
Control	Granulocytes	12.09%	665
Control	MDSC	30.30%	1667
Control	Monocyte	7.27%	400
Control	NK_Cell	1.96%	108
Control	Plasma_Cell	2.00%	110
Control	Proliferating_Granulocyte_Progenitor	10.71%	589
Control	T_Cell	8.52%	469

### B Cells in Mice Fracture Models

**Figure 3:**
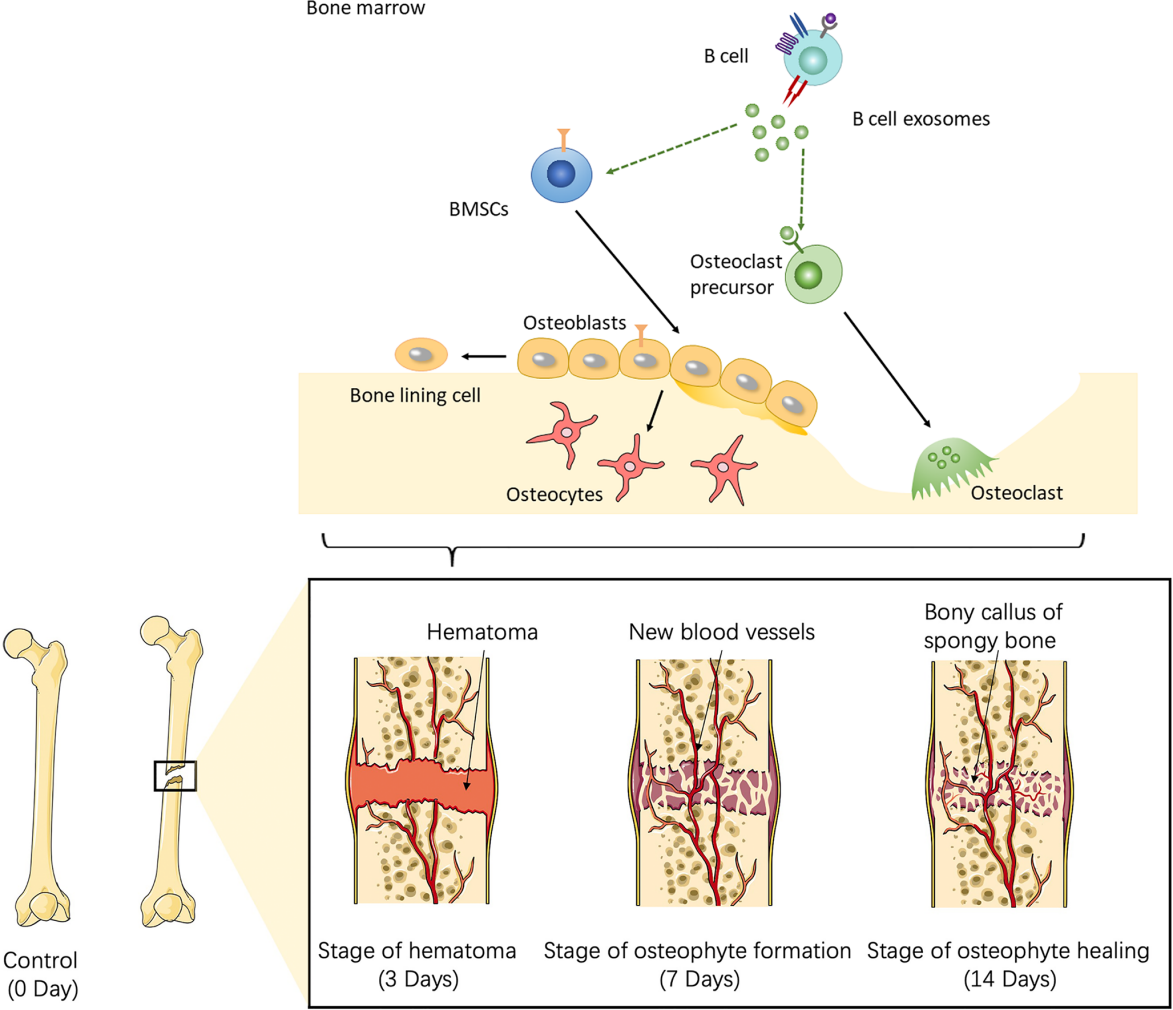
Schematic of the working hypothesis. B cells are consumed to the lowest level *via* inflammation during the hematoma stage, which promotes the recruitment of mesenchymal stem cells (MSCs) into osteogenic differentiation and angiogenesis. During the osteophyte formation stage, osteoblasts promote the maturation of B cells, which then inhibit the differentiation of osteoblasts and the abnormal proliferation of osteophytes. B cells can also promote the differentiation of osteoclasts and allow the body to enter the osteophyte healing stage. During osteophyte healing, osteoblasts and vascular endothelial cells decrease because of cytokines secreted by B cells, which then gradually return to normal levels. To summarize, B cells are a key regulator of fracture healing and inhibit excessive bone regeneration by producing multiple osteoblast inhibitors.

**Figure 4:**
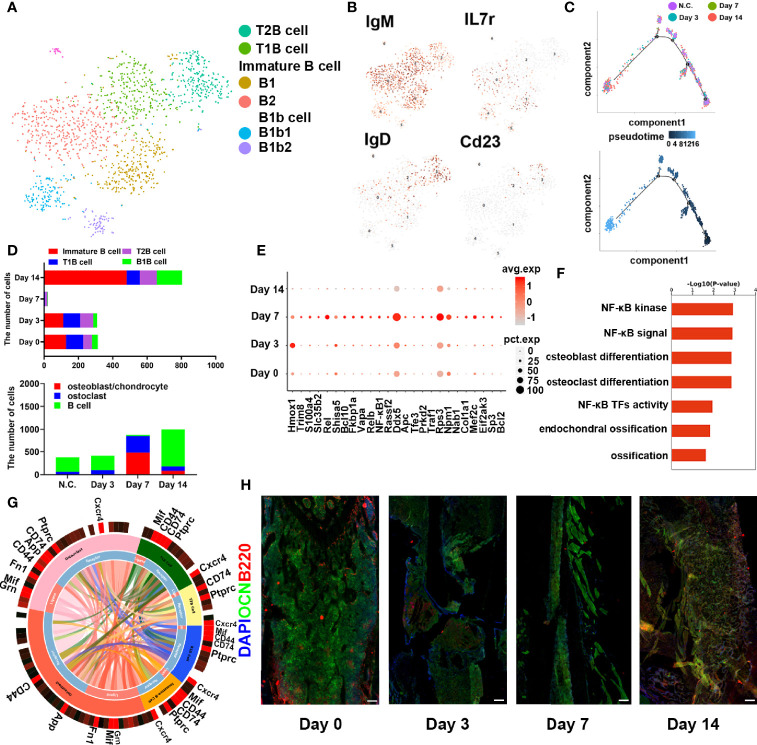
B cells in fracture models in mice. **(A)** t-SNE visualization of B cell subpopulations in mice fracture models. **(B)** Dot plots showing the expression of markers for each cell cluster in the B cell cluster. **(C)** Monocle2 pseudotime trajectory of the differentiation of B cells in mice fracture models. **(D)** Composition and proportion of B cells in the different periods in mice fracture models (control, day 0; hematoma stage, day 3; osteophyte formation stage, day 7; osteophyte healing stage, day 14). **(E)** Heat map of the scaled expression of differentially expressed genes related to fracture healing in the different periods in mice fracture models. **(F)** GO enrichment analysis of differentially expressed genes in the different periods in mice fracture models. **(G)** Cell phone communications between B cells and osteoblasts and osteoclasts. **(H)** Representative immunostaining images of B220 in B cells (red) and osteocalcin in osteoblasts (OCN, green). Scale bar: 100 μm.

To further investigate the role of B cells in fracture healing *in vivo*, mice fracture models were generated, and cells within 0.5 mm of the fracture were isolated at different stages (control group and on days 3, 7, and 14) (**Figure 3**). The B cells cluster was reclustered into six subclusters (**Figure 4A**), including T2B and T1B cells, immature B cells (B1 and B2), and B1b cells (B1b1 and B1b2), and the differentially expressed genes (DEGs) in the six subclusters were identified (**Figure 4B**). Consistent with the results in patients, the pseudotime trajectory axis indicated that the fewest B cells occurred in the callus formation stage, compared with those in the other stage of fracture healing and the normal bone marrow microenvironment (**Figure 4C**, **Tables 2**, **3**). By contrast, the callus healing stage had the most B cells. Furthermore, the bone formation and bone resorption activities in the mice fracture models were the most active in the osteophyte formation stage (day 7), compared with those processes in the other stage of fracture healing. However, in the osteophyte healing stage (day 14), bone formation and resorption activities decreased (**Figure 4D**). These results indicated that the decrease in B cells promoted osteoblast and osteoclast differentiation during callus formation, whereas the increase during callus healing inhibited osteoblast and osteoclast differentiation.

**Table 2:** The number of B cells in the case group and the control group.

Sample	CellType	Percentage	Number
Case	B Cell	30.40%	38
Case	pDC	26.40%	33
Case	Plasma Cell	43.20%	54
Control	B Cell	49.06%	131
Control	pDC	10.11%	27
Control	Plasma Cell	40.82%	109

**Table 3:** The number of B cells in the control, Day 3, Day 7, Day 14 groups.

**Sample**	**Celltype**	**Percentage**	**Number**
0Day	B1B Cell	10.86%	34
0Day	Immature B Cell	40.58%	127
0Day	T1B Cell	32.27%	101
0Day	T2B Cell	16.29%	51
3Day	B1B Cell	7.44%	23
3Day	Immature B Cell	35.92%	111
3Day	T1B Cell	32.36%	100
3Day	T2B Cell	24.27%	75
7Day	B1B Cell	9.09%	2
7Day	Immature B Cell	4.55%	1
7Day	T1B Cell	18.18%	4
7Day	T2B Cell	68.18%	15
14Day	B1B Cell	18.28%	147
14Day	Immature B Cell	59.83%	481
14Day	T1B Cell	9.45%	76
14Day	T2B Cell	12.44%	100

Moreover, DEGs related to osteogenesis were highly expressed during callus formation (**Figure 4E**). Gene Ontology analysis demonstrated that the set of DEGs in the osteophyte healing stage was involved in bone development and ossification (**Figure 4F**). Cell phone communication also showed that B cells regulated the differentiation of osteoblasts, osteoclasts (**Figure 4G**, **Figure S4**). Immunofluorescence further verified the changes in the numbers and percentages of B cells during fracture healing (**Figure 4H**) and showed the fewest B cells occurred on day 7. Thus, the scRNA-seq revealed that B cells might play an important role in fracture healing.

### B Cell Exosomes Inhibited Osteoblast Differentiation and Promoted Osteoclast Formation *In Vitro*

**Figure 5:**
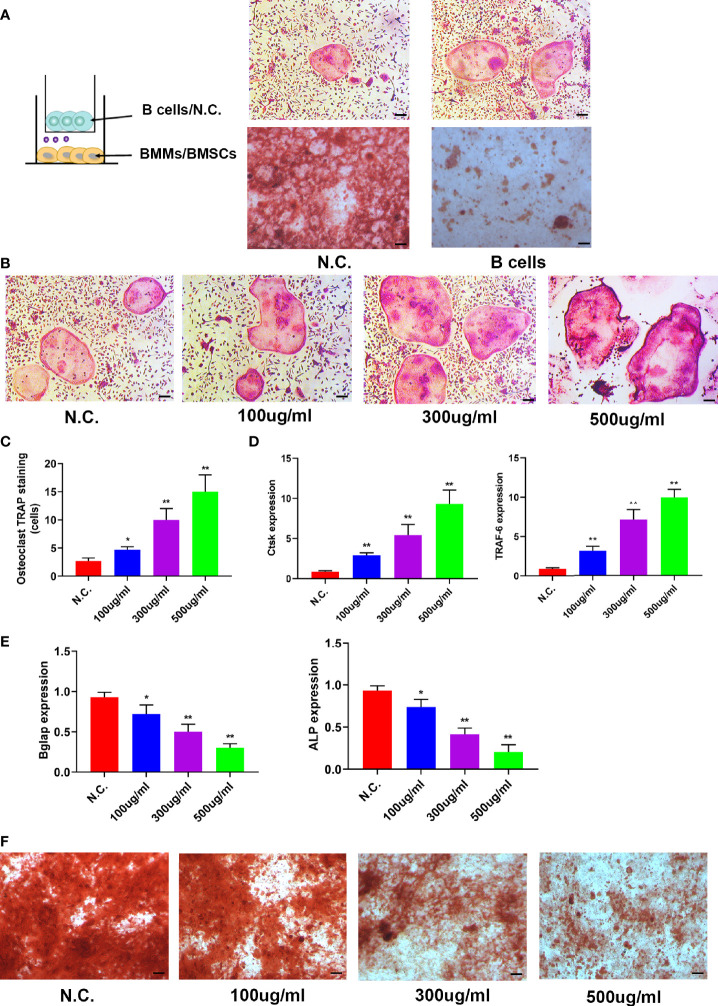
B cell exosomes (BC-Exos) inhibit osteoblast differentiation and promote osteoclast formation *in vitro*. **(A)** Representative images of TRAP-stained osteoclasts (top) and Alizarin red S-stained osteoblasts (bottom). Scale bar: 100 μm. **(B)** Representative images of TRAP-stained osteoclasts treated with different concentrations of BC-Exos. Scale bar: 100 μm. **(C)** Quantitative analysis of the cells of TRAP-stained osteoclasts in **(B)** (*p < 0.05, **p < 0.01). **(D)** Reverse-transcription qPCR (RT-qPCR) of the relative levels of Ctsk (left) and TRAF-6 (right) mRNA expression in osteoclasts in **(B)**. **(E)** RT-qPCR of the relative levels of Bglap (left) and ALP (right) mRNA expression in BMSCs at day 21. Data are reported as the mean ± SD of three independent experiments. **(F)** Representative images of Alizarin red S staining in osteoblasts at day 21. Scale bar: 100 μm.

To investigate the role of B cells in skeletal biology, whether B cells could affect osteoblasts or osteoclasts was determined. BMSCs or BMMs were co-cultured with BaF cells in a transwell system, and osteoclastic differentiation increased and osteogenic differentiation decreased when co-cultured with B cells (**Figure 5A**). Then, the exosomes derived from BaF cells (BC-Exos) were extracted and characterized. The BC-Exos had a cup-like morphology ranging from 70 to 150 nm (**Figures S3A, S3B**) and expressed CD63 and CD9 with Western blot analysis (**Figure S3C**).

To confirm the role of BC-Exos in bone homeostasis *in vitro*, mouse bone marrow monocytes were treated with BC-Exos or an equal volume of PBS. Osteoclastic differentiation increased as the concentration of BC-Exos increased (**Figures 5B, C**). Furthermore, Osteoclast differentiation marker genes Ctsk and DC-STAMP mRNA expression levels were upregulated by BC-Exos, compared with their negative controls at day 7 (**Figure 5D**). Moreover, pre-osteoblast MC3T3-E1 cells were also treated with BC-Exos or an equal volume of PBS *in vitro*. Osteoblast maturation marker genes BGLAP and Alp mRNA expression levels were downregulated by BC-Exos at day 21 (**Figure 5E**). Consistent with the results of BGLAP and Alp expression, mineral deposition in BC-Exos-treated cells decreased, compared with that in the negative control in pre-osteoblast MC3T3-E1 cells (**Figure 5F**). Thus, these data suggested that BC-Exos inhibited osteoblast differentiation and promoted osteoclast formation *in vitro*.

### B Cell Exosomes Inhibited Bone Formation *In Vivo*

**Figure 6:**
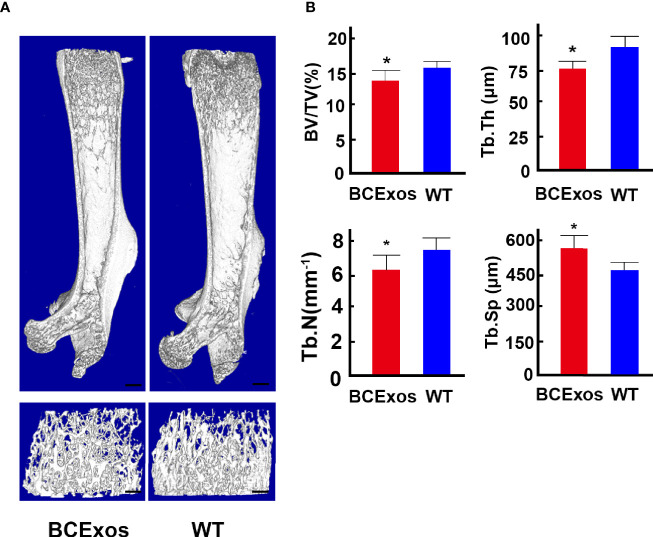
B cell exosomes (BC-Exos) inhibit bone formation *in vivo*. **(A)** Representative microcomputed tomography images in femora from BC-Exos mice and their controls (*n* = 7 per group). Scale bar: 100 μm. **(B)** Quantitative microcomputed tomography analysis of trabecular bone microarchitecture in femora of BC-Exos mice and their controls. BV/TV, trabecular bone volume per tissue volume; Tb. Th, trabecular thickness; Tb. Sp, trabecular separation; Tb. N, trabecular number (*n* = 7 per group). *p < 0.05.

To determine the role of BC-Exos *in vivo*, BC-Exos or an equal volume of PBS was injected into the tail vein of 8-week-old male mice twice per week for eight weeks. Microcomputed tomography (μ-CT) showed significantly lower trabecular bone volume, trabecular number, and trabecular thickness and higher trabecular separation in mice injected with BC-Exos than in their controls (**Figures 6A, B**).

## Discussion

In this study, immune cells in the bone marrow microenvironment were investigated at single-cell resolution in different stages of fracture healing. There were fewer B cells in old fracture tissues than in fresh fracture tissues. In addition, the callus formation stage had the fewest B cells, in contrast to the callus healing stage with the most. These results suggest that B cells have an important role in fracture healing. The B cell exosomes were characterized. Furthermore, B cell exosomes inhibited osteoblast differentiation and promoted osteoclast formation *in vitro*, and when injected in mice, osteogenic activity decreased significantly. In conclusion, B cell exosomes could regulate osteoblast and osteoclast differentiation and this might be used as a nano-medicine to treat diseases such as fractures or osteoporosis.

B cells are involved in the pathogenesis of rheumatic diseases, including rheumatoid arthritis, Vasculitis (25), and multiple sclerosis (26, 27), which are related to anti-neutrophil cytoplasmic antibodies through B cell intrinsic, antibody-mediated (28), and T cell-dependent mechanisms (29). Furthermore, dysregulated B cell expression of RANKL and OPG correlates with loss of bone mineral density in HIV infection (30). However, the relations between B cells and fracture-related disease remain unclear. In this study, B cells were confirmed as regulators of fracture healing. In the early stage of callus formation, differentiation of osteoblasts and osteoclasts increased with a decrease in the number of B cells. During the callus healing stage, excessive osteoblast differentiation was inhibited with increasing numbers of B cells, which implied that that changes in the number of B cells in the bone marrow microenvironment might cause bone loss. Furthermore, Btk has been reported to regulate osteoclast differentiation by RANK and ITAM signals, which congenital defect can cause an arrest in B cell development and immunodeficiency (31).This indicated that it might lead to insufficient RANK signal which in turn leads to a decrease in the differentiation of osteoclasts or osteoblasts. In our study, we found that B cell exosomes could regulate osteoblast and osteoclast differentiation, which can be used as a nanomedicine to treat fractures or osteoporosis. Therefore, this work should stimulate future works to investigate the correlation between bone formation and B cell development, which could lead to potential new therapeutic targets.

Single-cell RNA sequencing has recently revolutionized study of the bone marrow microenvironment and its related diseases (32, 33). The heterogeneity of pathogenic T-helper 17 cells was determined by combining scRNA-seq and mouse experimental autoimmune encephalomyelitis models (34). In addition, scRNA-seq was used to analyze tumor-infiltrating T cells and revealed exhaustion programs in human metastatic melanoma (35). However, research that combines scRNA-seq with exosomes remains rare. In this study, MC3T3-E1 cells or BMMs were treated with BC-Exos based on scRNA-seq results. The BC-Exos inhibited osteoblast differentiation and promoted osteoclast formation *in vitro*, and thus, these results expanded the application of single-cell technology. Furthermore, the interactions between different cells in the bone marrow microenvironment were identified *via* cell phone bioinformatics analysis, which helped to understand the importance of those interactions. These results also provide a basis to increase understanding for the safe use of exosomes in the targeted delivery of biological nanomaterials.

## Data Availability Statement

The data presented in the study are deposited in the GEO dataset repository, accession number (GSE142786 and GSE132884).

## Ethics Statement

The animal study was reviewed and approved by Scientific Investigation Board of Second Military Medical University.

## Author Contributions

RW and HZ contributed to the conception and design of this study, the performance of experiments, interpretation, data analysis, and manuscript writing. BZ and ZF performed data analysis and interpretation. HT and FJ contributed to the design of this study, acquiring financial support, data analysis, interpretation, manuscript writing, and the final approval of the manuscript. All authors contributed to the article and approved the submitted version.

## Funding

The National Natural Science Foundation of China supported this study (nos. 81572637, 81702666, 81272942, 81202122, and 30973019).

## Conflict of Interest

BZ and CW were employed by the company Novel Bioinformatics Ltd., Co. Shanghai, China.

The remaining authors declare that the research was conducted in the absence of any commercial or financial relationships that could be construed as a potential conflict of interest.

## Publisher’s Note

All claims expressed in this article are solely those of the authors and do not necessarily represent those of their affiliated organizations, or those of the publisher, the editors and the reviewers. Any product that may be evaluated in this article, or claim that may be made by its manufacturer, is not guaranteed or endorsed by the publisher.
