# Supplementary material for: Single-Cell RNA Sequencing Reveals B Cells Are Important Regulators in Fracture Healing
Source: Front Endocrinol (Lausanne). 2021 Nov 8;12:666140. doi: 10.3389/fendo.2021.666140 (PMC8606664; doi:10.3389/fendo.2021.666140)
Supplement: Supplementary file 1 [file DataSheet_1.docx]

**
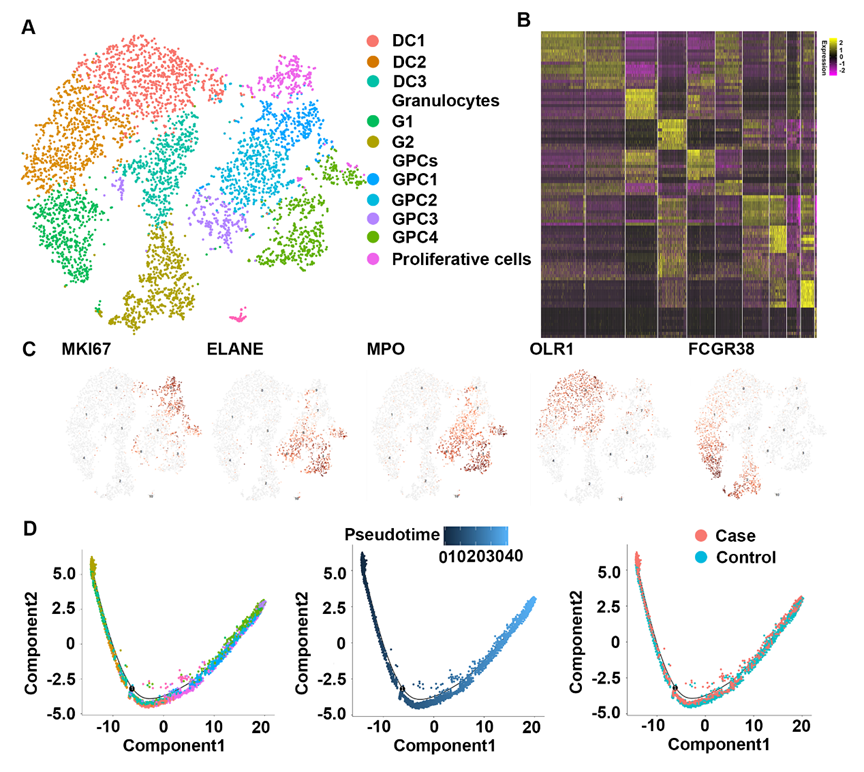
**

**Figure S1 Identification of neutrophils.** (A) t-SNE visualization of neutrophil subpopulations. (B) Heatmap revealing the scaled expression of differentially expressed genes for each cluster defined in neutrophil subpopulations. (C) Dot plots showing the expression of indicated markers for each cell cluster in neutrophil subpopulations. (D) Monocle pseudotime trajectory revealing the differentiation of neutrophil subpopulations.

**
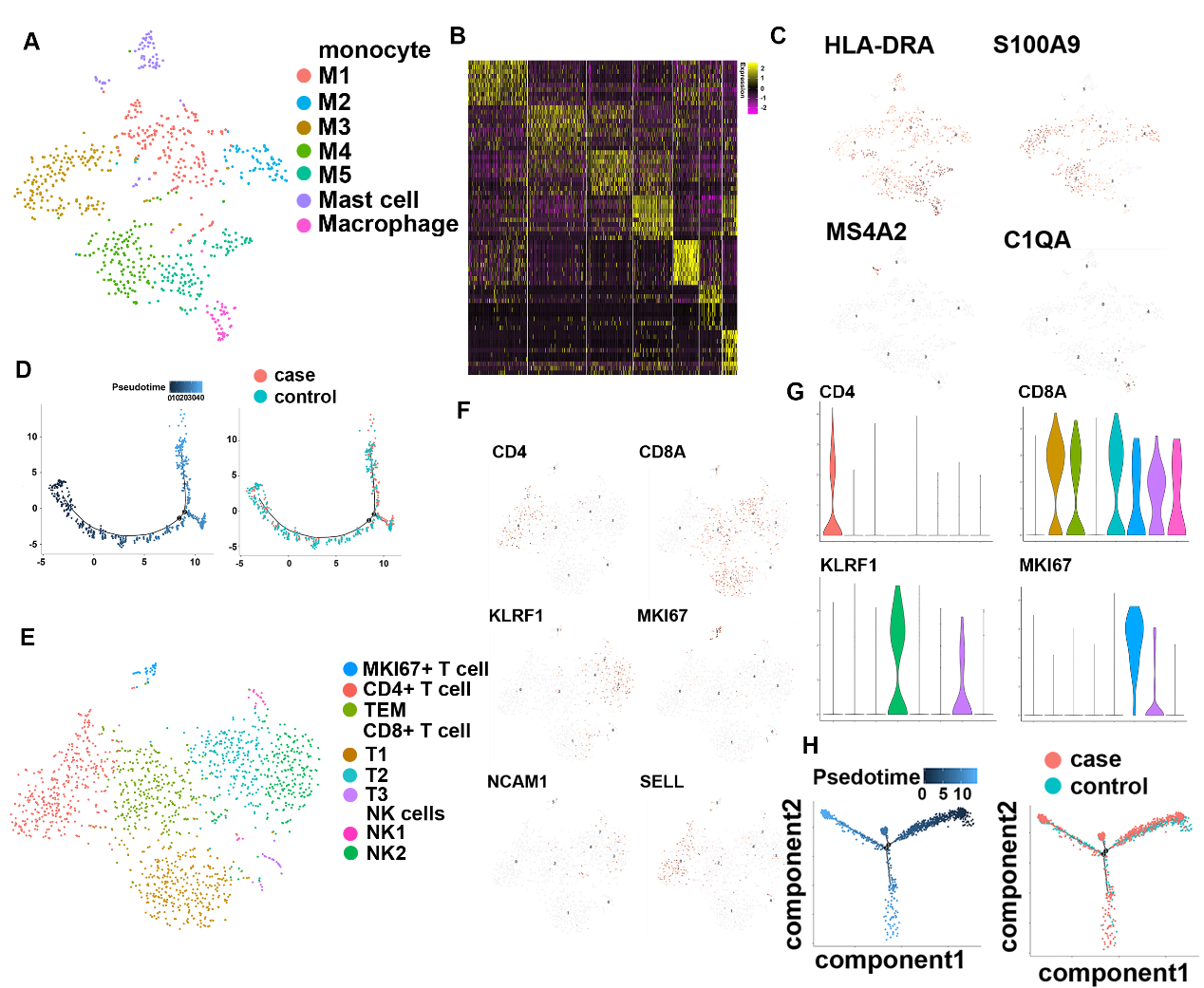
**

**Figure S2 Identification of monocytes and T cells.** (A) t-SNE visualization of monocytes subpopulations. (B) Heatmap revealing the scaled expression of differentially expressed genes for each cluster defined in monocytes subpopulations. (C) Dot plots showing the expression of indicated markers for each cell cluster in monocyte clusters. (D) Monocle pseudotime trajectory revealing the differentiation of monocytes. (E) t-SNE visualization of T cells subpopulations. (F) Dot plots showing the expression of indicated markers for each cell cluster in T cells clusters. (G) Violin plots showing the expression levels of representative T cell markers for T cells clusters. (H) Monocle pseudotime trajectory revealing the differentiation of T cells.

**
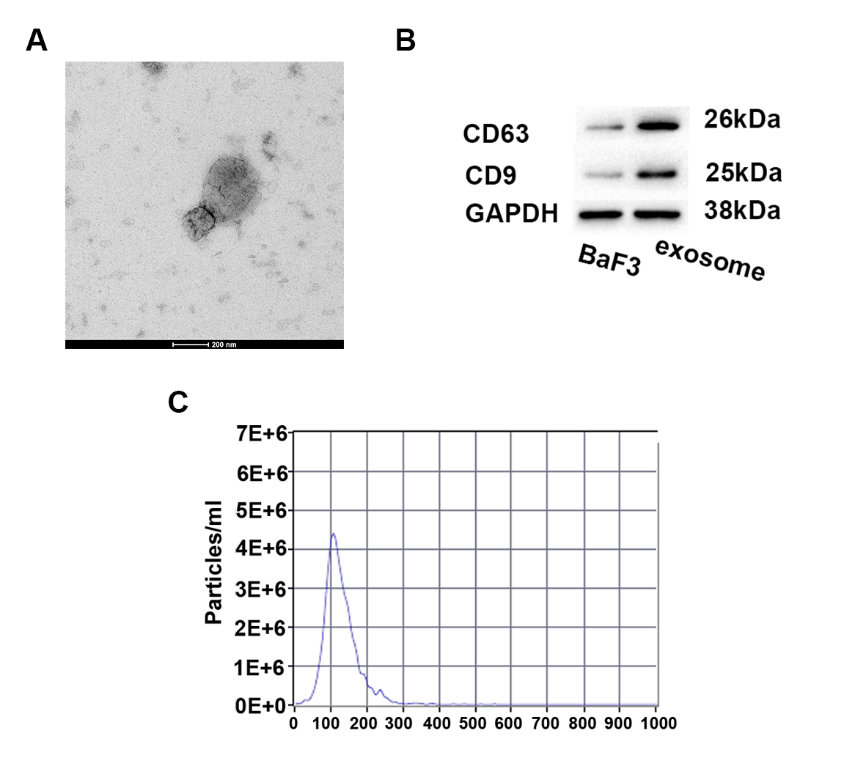
**

**Figure S3 Characteristics of BC-Exos.** (A) Representative TEM image of BC-Exos (scale bar = 200 nm). (B) Representative NTA results showing the size distribution of BC-Exos. (C) Detection of BC-Exos surface markers (CD63, CD9) by Western Blot.


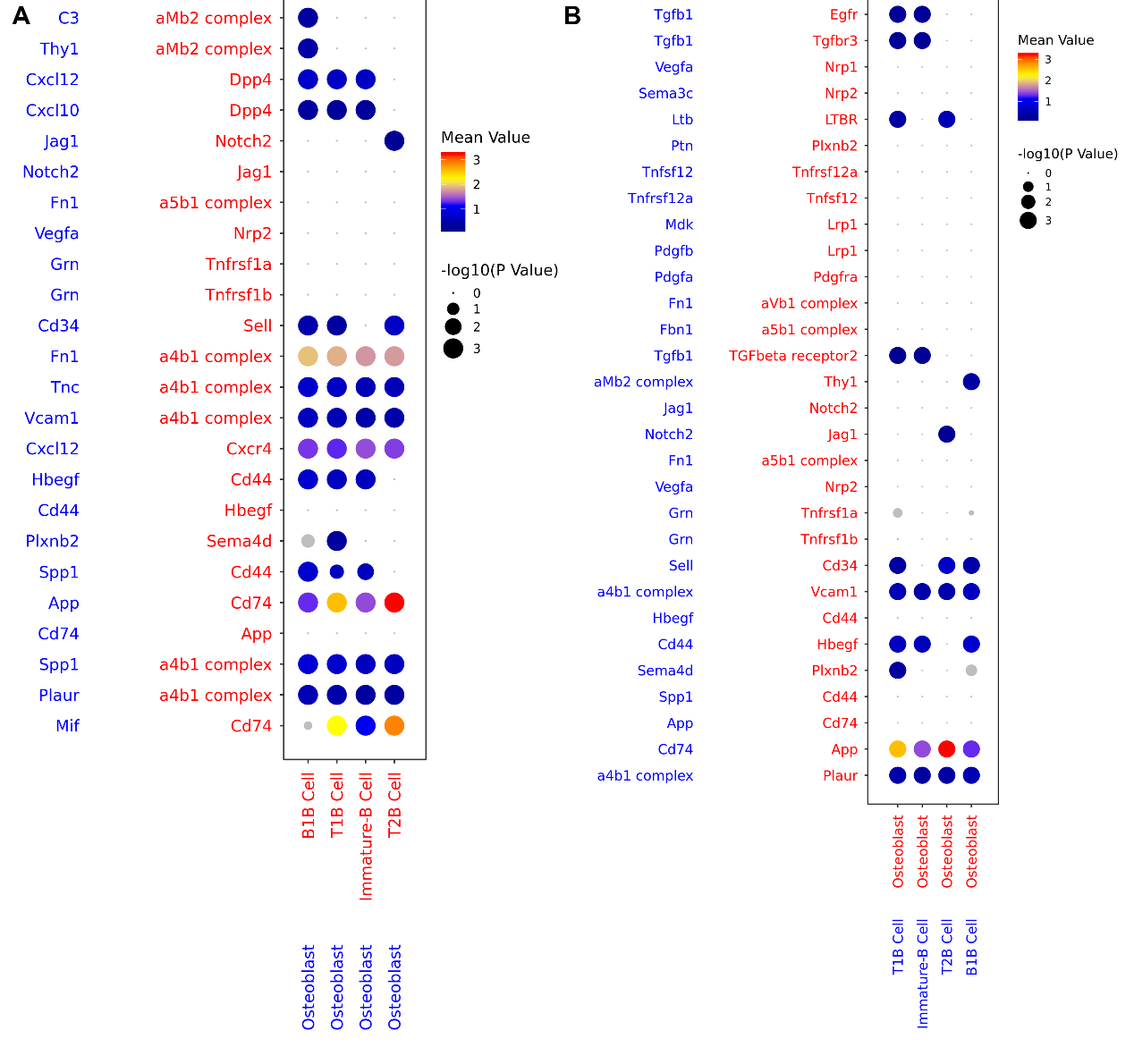


**Figure S4 Cell phone communications between B cells and osteoblasts.**
